# An Unsupervised Change Detection Method Using Time-Series of PolSAR Images from Radarsat-2 and GaoFen-3

**DOI:** 10.3390/s18020559

**Published:** 2018-02-12

**Authors:** Wensong Liu, Jie Yang, Jinqi Zhao, Hongtao Shi, Le Yang

**Affiliations:** State Key Laboratory of Information Engineering in Surveying, Mapping and Remote Sensing, Wuhan University, Wuhan 430079, China; liuwensong@whu.edu.cn (W.L.); masurq@whu.edu.cn (J.Z.); sht9010@whu.edu.cn (H.S.); yang.le@whu.edu.cn (L.Y.)

**Keywords:** time-series, unsupervised change detection, PolSAR, omnibus test statistic, GSRM, GGMM

## Abstract

The traditional unsupervised change detection methods based on the pixel level can only detect the changes between two different times with same sensor, and the results are easily affected by speckle noise. In this paper, a novel method is proposed to detect change based on time-series data from different sensors. Firstly, the overall difference image of the time-series PolSAR is calculated by omnibus test statistics, and difference images between any two images in different times are acquired by *R_j_* test statistics. Secondly, the difference images are segmented with a Generalized Statistical Region Merging (GSRM) algorithm which can suppress the effect of speckle noise. Generalized Gaussian Mixture Model (GGMM) is then used to obtain the time-series change detection maps in the final step of the proposed method. To verify the effectiveness of the proposed method, we carried out the experiment of change detection using time-series PolSAR images acquired by Radarsat-2 and Gaofen-3 over the city of Wuhan, in China. Results show that the proposed method can not only detect the time-series change from different sensors, but it can also better suppress the influence of speckle noise and improve the overall accuracy and Kappa coefficient.

## 1. Introduction

The successful launch of China’s first multi-polarization synthetic aperture radar (SAR) imaging satellite (Gaofen-3, GF3) on 10 August 2016 [1], has greatly promoted research on PolSAR in China [2]. The GF3 data possess not only the advantages of the traditional SAR images, such as being immune to the influence of weather and illumination, but they also feature a variety of polarization imaging modes, allowing us to obtain more information on the scattering of objects and achieve improved object interpretation [3]. With the development of PolSAR satellites, a large number of time-series PolSAR images are now available from different sensors (such as ENVISAT-ASAR, ALOS-PALSAR, TerraSAR-X, Radarsat-2), which can better reflect the dynamic changes of the Earth’s surface. These images have been used in a wide range of applications in the fields of disaster prevention and mitigation [4,5,6], agriculture monitoring [7], forestry [8], land-cover change [9,10] and weather forecasting [11]. Therefore, research on change detection with PolSAR time-series images is of great significance and has aroused widespread interest [12,13,14]. 

Although the traditional pixel-based unsupervised change detection methods for PolSAR images can detect the change between two different times with the same sensor (see Table 1 for a summary of advantages and disadvantages using traditional unsupervised change-detection methods [15,16]), they cannot detect the total change of a time series from different sensors [17]. To solve these problems, some researchers have simply compared pair-wise images in the time-series images and detected the time-series change, which suffers from some deficiencies, i.e., it is time-consuming and cannot detect some small, continuous changes [17]. On the other hand, the results of traditional pixel-based unsupervised change detection methods are susceptible to speckle noise [18], which can result in a high false alarm rate. In recent years, to address these issue, researchers have proposed object-oriented change detection methods that use a segmentation algorithm to segment the PolSAR images from two different times, and then used the traditional pixel-based method to detect the change [19,20]. Although these methods can reduce the effect of speckle noise, they cannot maintain the consistency of segmentation. When analyzing the difference images, traditional methods such as Two-Dimensional Entropic Segmentation (TDES) algorithm [21], Otsu’s thresholding algorithm [22], improved Kittler and Illingworth (K&I) algorithm [23] and Kapur’s entropy algorithm [24], assume that the Probability Density Function (PDF) of the difference image complies with a Gaussian distribution. However, the difference images calculated by omnibus test statistic and *R_j_* test statistic algorithm do not comply with a Gaussian distribution [17]. As a consequence, traditional algorithms are not suitable for the analysis of difference image which obtained by omnibus or *R_j_* test statistic algorithms. Fortunately, Gaussian Mixture Models (GMM) can fit any distribution of data [25]. In our previous work [26], by improving the GMM algorithm, we are better able to analyze image differences.

In this paper, we expand on our previous work in time-series analysis and present an unsupervised change detection method (named OT_GSRM_GGMM) for different sensors. Firstly, the overall difference image of the time-series PolSAR images is calculated by omnibus test statistics, and the difference images between any two images in different times are acquired by *R_j_* test statistics. Secondly, the difference images are segmented with a GSRM algorithm which can suppress the effect of speckle noise. GGMM is used to obtain time-series change detection maps in the last step for the proposed method. Since the city of Wuhan in China has undergone tremendous changes during the ‘Twelfth Five-Year Plan‘ period [27], the proposed method of this paper was used to detect the changes in Wuhan’s urban development from 2011 to 2017. 

This paper is organized as follows: in Section 2, the framework of time-series change detection is described and the methods of omnibus test statistic, GSRM and GGMM techniques are introduced. Section 3 details the results of the proposed approach on time-series PolSAR images from the city of Wuhan. Section 4 discusses the results of the case study. Finally, the conclusions are drawn in Section 5.

## 2. Materials and Methods

### 2.1. Omnibus Test Statistic

The omnibus test statistic algorithm effectively utilizes polarimetric and temporal information from time-series PolSAR images. A PolSAR image includes the backscattering coefficients of the four polarimetric channels of the object [28]. For orthogonal polarization basis, the scattering information of ground objects can be represented by the following covariance matrix *C*:

(1) $$C=\langle \left[\begin{array}{ccc} {\left|S_{hh}\right|}^{2} & S_{hh}S_{hv}^{*} & S_{hh}S_{vv}^{*}\\ S_{hv}S_{hh}^{*} & {\left|S_{hv}\right|}^{2} & S_{hv}S_{vv}^{*}\\ S_{vv}S_{hh}^{*} & S_{vv}S_{hv}^{*} & {\left|S_{vv}\right|}^{2} \end{array}\right]\rangle$$

Different elements in covariance matrix *C* represent the backscattering coefficients. For the multi-look conditions, the covariance matrix *C* of a PolSAR image obeys the complex Wishart distribution ($X\in W(p,n,\Sigma_{X})$) and PDF of *C* can be described as follows:

(2) $$\begin{array}{l} f(C)=\frac{1}{\Gamma_{n}(p)}\frac{1}{{\left|\Sigma_{C}\right|}^{n}}{\left|C\right|}^{n-p}\exp\{-tr[\Sigma_{C}^{-1}C]\}\\ \Gamma_{p}(n)=\pi^{p(p-1)/2}\prod_{j=1}^{p}\Gamma (n-j+1) \end{array}$$

where, *tr*(·) is the trace of covariance matrix *C*, n is the number of looks of PolSAR image, and *p* represents the dimension of matrix *C*. For fully a PolSAR image, p is equals to 3 [17].

We assume that the multi-parameters $\sum_{X_{1}},\sum_{X_{2}},\ldots \sum_{X_{j-1}},\sum_{X_{j}},\ldots \sum_{X_{k}}$ of time-series ($t_{1}<t_{2}<\ldots <t_{k}$) PolSAR images are independent, and they obey the complex Wishart distribution:

(3) $$\begin{array}{l} X_{1}\in W(p,n_{1},\Sigma_{X_{1}})\\ X_{2}\in W(p,n_{2},\Sigma_{X_{2}})\\ \begin{array}{ccc} & \cdots & \end{array}\\ X_{k}\in W(p,n_{k},\Sigma_{X_{k}}) \end{array}$$

where p represents the dimension of $X_{1},X_{2},\ldots ,X_{k}$, $n_{1},n_{2},\ldots ,n_{k}$ is the number of look of $X_{1},X_{2},\ldots ,X_{k}$, and $\Sigma_{X_{1}},\Sigma_{X_{2}},\ldots ,\Sigma_{X_{k}}$ represent the scattering matrix of $X_{1},X_{2},\ldots ,X_{k}$.

According to omnibus test statistic theory, the *H*_0_ hypothesis can be described as $H_{0}:\sum_{X_{1}}=\sum_{X_{2}}=\ldots =\sum_{X_{j-1}}=\sum_{X_{j}}=\ldots \sum_{X_{k}}$, which means the matrices of time-series PolSAR images are equal. In other words, if *H*_0_ hypothesis were the case, the feature has not changed in the time interval [$t_{1}$, $t_{k}$]. If not, the feature has at least one change in the time-series [$t_{1}$, $t_{k}$] of PolSAR images.

We supposing that the omnibus test statistic based on maximum likelihood estimation (MLE) has a joint density $f(\sum_{X_{1}},\sum_{X_{2}}\ldots \sum_{X_{k}},\theta )$, where θ is the set of parameters of the probability function that has generated the data. *H*_0_ states that $\theta \in H_{0}$, and the likelihood ratio of the omnibus test statistic is as follows:

(4) $$Q=\frac{\max_{\theta \in H_{0}}L(\theta )}{\max_{\theta \in \Omega }L(\theta )}=\frac{L(\sum_{S})}{\prod_{i=1}^{i=k}L_{X_{1}}(\Sigma_{X_{i}})}$$

where:

(5) $$\prod_{i=1}^{i=k}L_{X_{i}}(\Sigma_{X_{i}})=\frac{1}{\prod_{i=1}^{i=k}\Gamma_{p}(n_{i})}\prod_{i=1}^{i=k}{\left|\Sigma_{X_{i}}\right|}^{-n_{i}}\prod_{i=1}^{i=k}{\left|\Sigma_{X_{i}}\right|}^{n_{i}-p}\exp\{-tr(\sum_{i=1}^{i=k}\Sigma_{X_{i}}^{-1}X_{i})\}\;L(\Sigma_{S})=\frac{1}{\prod_{i=1}^{i=k}\Gamma_{p}(n_{i})}{\left|\Sigma \right|}^{-\sum_{i=1}^{i=k}n_{k}}\prod_{i=1}^{i=k}{\left|\Sigma_{X_{i}}\right|}^{n_{i}-p}\exp\{-tr(\Sigma^{-1}\left|X\right|)\}$$

If $n_{1}=n_{2}=\cdots =n_{k}=n$, this leads to the desired likelihood-ratio omnibus test statistic [29]:

(6) $$Q=\frac{\max_{\theta \in H_{0}}L(\theta )}{\max_{\theta \in \Omega }L(\theta )}=\frac{L(\sum_{S})}{\prod_{i=1}^{i=k}L_{X_{1}}(\Sigma_{X_{i}})}={\{k^{pk}\frac{\prod_{i=1}^{i=k}\left|X_{i}\right|}{{\left|X\right|}^{k}}\}}^{n}$$

where, $X=\sum_{i=1}^{i=k}X_{i}$, $X_{i}=n<C{>}_{i}$ and Equation (6) in logarithmic form is as follows:

(7) $$lnQ=n\{pklnk+\sum_{i=1}^{i=k}ln\left|X_{i}\right|-kln\left|X\right|\}$$

In general, the overall similarity of time-series PolSAR images is measured by −lnQ. The larger the value, the greater the probability that change will occur in time-series PolSAR images.

### 2.2. R_j_ Test Statistic

The omnibus test statistic algorithm can be used to detect the overall change of the time-series PolSAR images, but it is limited to detecting the change between any two different times. To compensate for the shortcoming of omnibus test statistic, Conradsen et al. formulated the *R_j_* test statistic algorithm, which is used to generate the different images in different time intervals [17]. According to *R_j_* test statistics, if the matrices of any two different PolSAR images in time are equal ($H_{0}:\sum_{X_{j-1}}=\sum_{X_{j}}$), it indicates that there is no change in the time interval [$t_{j-1}$, $t_{j}$]. Instead, if the matrices are not equal ($H_{1}:\sum_{X_{j-1}}\neq \sum_{X_{j}}$), the change happens between the two images. According to *R_j_* test statistics, the likelihood ratio of the statistic can be shown as follows:

(8) $$\begin{array}{cc} R_{j} & =\frac{j^{jpn}}{{\left(j-1\right)}^{(j-1)pn}}\frac{{\left|X_{1}+\ldots +X_{j-1}\right|}^{(j-1)n}{\left|X_{j}\right|}^{n}}{{\left|X_{1}+\ldots +X_{j}\right|}^{jn}} \end{array}{\begin{array}{cc} & =\left\{\frac{j^{jp}}{{\left(j-1\right)}^{(j-1)p}}\frac{{\left|X_{1}+\ldots X_{j-1}\right|}^{\left(j-1\right)}}{{\left|X_{1}+\ldots X_{j}\right|}^{j}}\right\} \end{array}}^{n}$$

Equation (8) in logarithmic form is as follows:

(9) $$lnR_{j}=n\{p(jlnj-(j-1)ln(j-1))+(j-1)ln\left|\sum_{i=1}^{j-1}X_{i}\right|+ln\left|X_{j}\right|-jln\left|\sum_{i=1}^{j}X_{i}\right|$$

Similarly, the similarity of PolSAR images from two any different times is measured by $-lnR_{j}$. The larger the value, the greater the probability that change will occur between the two images.

### 2.3. Generalized Statistical Region Merging (GSRM)

PolSAR data are often seriously affected by multiplicative speckle noise. Even with filter processing, the results obtained with traditional pixel-based change detection methods are still affected by speckle noise. Thus, object-oriented change detection algorithm was proposed to suppress the influence of speckle noise [29] and its core is segmentation.

The Statistical Region Merging (SRM) algorithm, with its small computation burden, is independent of the statistical characteristics of data. What is more, it has a strong capability of speckle noise immunity, when compared with the super-pixel segmentation algorithm, such as Ncut segmentation [30] and Mean Shift algorithm [31]. However, SRM was originally used in optical image processing with a range of [0, 255], and it cannot be used to segment PolSAR images whose numerical range was not fixed. Fortunately, Lang et al. extended SRM and proposed the generalized SRM (GSRM) algorithm [32].

GSRM algorithm defines two necessary elements: the merging criteria and merging order. Merging criteria indicates that if two adjacent regions in PolSAR image meet a certain condition, the two regions are merged. According to the martingale theory in probability theory, for two adjacent regions (R,R′) in PolSAR image *I*, there is:

(10) $$\mathrm{Pr}\left(\left|\left(\bar{R}-\bar{R\prime }\right)-E\left(\bar{R}-\bar{R\prime }\right)\right|\geq \sqrt{\frac{B^{2}}{2Q}\left(\frac{E^{2}\left(\bar{R}\right)}{\left|R\right|}+\frac{E^{2}\left(\bar{R\prime }\right)}{\left|R\prime \right|}\right)\ln\frac{2}{\delta }}\right)\leq \delta$$

where 0 < δ≤ 1. The two adjacent regions (R,R′) are merged under condition of Equation (11):

(11) $$\left|\bar{R}-\bar{R\prime }\right|\leq b(R,R\prime )$$

where:

(12) $$b(R,R\prime )=g\sqrt{\frac{1}{2Q}\left(\frac{1}{\left|R\right|}+\frac{1}{\left|R\prime \right|}\right)\ln\frac{2}{\delta }}$$

Merging order defines which two areas should be merged first when merging PolSAR images. GSRM algorithm adopts a pre-sorting strategy, where the adjacent pairs of pixels in the PolSAR image *I* are first sorted according to the gradient function f(p,p′). Gradient function defined is as follows:

(13) $$f(p,p\prime )={\left\|\frac{p-p\prime }{p+p\prime }\right\|}_{1}$$

### 2.4. Generalized Gaussian Mixture Model (GGMM)

GMM [25] is an algorithm that can fit any distribution of PDF. Therefore, it is widely used in remote sensing data interpretation, such as clustering and unsupervised change detection. Since the distribution of difference images obtained by GSRM is unknown, the GMM algorithm is more suitable for the analysis of difference images than the traditional algorithms, such as TDES, K&I and Otsu’s thresholding algorithm. Assuming that the number of Gaussian functions of GMM model is k and its expression can be given by Equation (14):

(14) $$f(x)=\sum_{i=1}^{k}\alpha_{i}p(x|\theta_{i})\;\theta =\left\{\mu_{1},..,\mu_{m},\sigma_{{}_{1}}^{2},\ldots ,\sigma_{{}_{m}}^{2}\right\}$$

where $\alpha_{i}$ is the weight of *i*th Gaussian function, total value of $\alpha_{i}$ equals 1, $\mu_{i}$ and $\sigma_{i}^{2}$ represent the mean and variance of *i*th Gaussian function, respectively. In the process of analysis, difference image can be described as sum of change class ($p(x|\theta_{c})$) and no-change class ($p(x|\theta_{u})$) as shown in Equation (15). Notably, $p(x|\theta_{u})$ and $p(x|\theta_{c})$ have different weights:

(15) $$p(x_{d})=\sum_{k=1}^{K}p(k)p(x_{d}|k)=\begin{array}{ccc} \sum_{\forall \theta_{u}\in M_{u}}p(\theta_{u})p(x|\theta_{u}) & + & \sum_{\forall \theta_{c}\in M_{c}}p(\theta_{c})p(x|\theta_{c}) \end{array}$$

where, $p(\theta_{u})$ and $p(\theta_{c})$ represent the weight of $p(x|\theta_{c})$ and $p(x|\theta_{u})$, respectively, $p(x|\theta_{u})$ and $p(x|\theta_{c})$ are the PDF of change class and no-change class. In general, the parameters of Equation (15) can be solved by log-likelihood function. However, the existence of unknown parameter θ makes this impossible.

The Expectation Maximization (EM) algorithm is a better solution to the model with unknown parameters in GMM [33]. The main idea of EM is to use an iterative method to calculate the weight, mean and variance of all Gaussian functions in GMM. To calculate the mean and variance of each Gaussian function, MLE is the best choice. EM method mainly contains two steps, one is expectation (E) step and another is maximization (M) step. E-step can estimate the parameters of $\alpha_{i}$, $\mu_{i}$ and $\sigma_{i}^{2}$ by iteratively operating with likelihood function (16):

(16) $$Q(\theta ,\hat{\theta }(t))\equiv E[\log p(X,\gamma |\theta )|X,\hat{\theta }(t)]=\log p(X,\hat{\gamma }|\theta )$$

where:

(17) $${\hat{\gamma }}_{im}=E[\gamma_{im}|x,\hat{\theta }(t)]=\frac{{\hat{\alpha }}_{m}(t)p(x_{i}|{\hat{\theta }}_{m}(t))}{\sum_{j=1}^{k}{\hat{\alpha }}_{j}(t)p(x_{i}|{\hat{\theta }}_{j}(t))}$$

M-step obtains the desired maximum with the derivation operation (Equation (18)) and parameters from E-step:

(18) $$\frac{\partial (Q(\theta ,\hat{\theta }(t)))}{\partial \theta }=0$$

Finally, according to the convergence condition of EM algorithm, the iterative updating of each unknown parameter is realized:

(19) $$\alpha_{i}=\sum_{i=1}^{n}{\hat{\gamma }}_{im}/n\;\mu_{i}=\sum_{i=1}^{n}{\hat{\gamma }}_{im}x_{i}/\sum_{i=1}^{n}{\hat{\gamma }}_{im}\;\sigma_{i}^{2}=\sum_{i=1}^{n}{\hat{\gamma }}_{im}{\left(x_{i}-\mu_{i}\right)}^{T}(x_{i}-\mu_{i})/d\sum_{i=1}^{n}{\hat{\gamma }}_{im}$$

Nevertheless, the parameter *k* (the number of Gaussian function) in traditional GMM algorithm is unknown. Researchers can only estimate the value of k using empirical parameter values or by iterative operating, which is time-consuming and gives unsatisfactory results [25]. Therefore, in our previous study [26], we improved the traditional GMM model and used the elbow method to obtain the optimal value of k in advance, which can solve the problem of the traditional GMM algorithm, where parameter k cannot be determined.

### 2.5. Quantitative Evaluation Criteria

It is very important to evaluate the results of proposed method using quantitative evaluation criteria. The False Alarm (*FA*) rate, Omission Factor (*OF*) rate, Overall Accuracy (*OA*) and Kappa coefficient (*Kappa*) [23] of the experimental results were calculated in this study and the quantitative evaluation criteria are calculated as follows:

(20) $$\left\{ \begin{array}{l} FA=\frac{FP}{N_{u}}\\ OF=\frac{FN}{N_{c}}\\ OA=\frac{TP+TN}{N}\\ Kappa=\frac{OA-Pe}{1-Pe}\\ Pe=\frac{\left(TP+FN)(TP+FP)+(FP+TN)(FN+TN\right)}{N^{2}} \end{array}\right.$$

where, *FP* means the number of unchanged points incorrectly detected as changed; *FN* means the number of changed points incorrectly detected as unchanged; *TP* means the number of changed points correctly detected; *TN* means the number of unchanged points correctly detected; *N_u_* and *N_c_* are the number of unchanged points and changed points of the ground-truth change map; and *N* is the sum of *N_u_* and *N_c_*, respectively.

### 2.6. The Proposed Method of Time Series Change Detection Using Images from Different Sensors 

**Figure 1:**
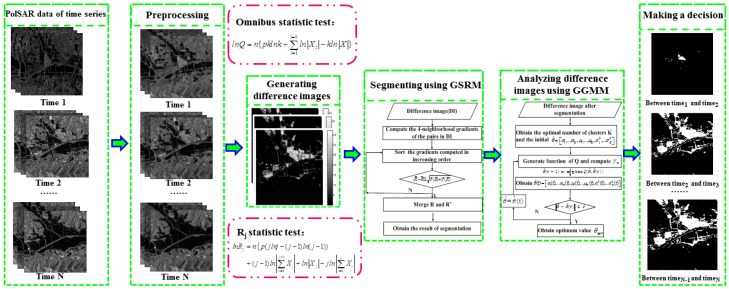
The procedure of the proposed method.

The procedure of time-series unsupervised change detection based on different sensors is shown in Figure 1. The process of change detection includes, (1) Data preprocessing, including radiometric correction, geometric correction, co-registration and filtering. (2) Calculating the overall difference image of a time-series image by omnibus test statistic and acquiring the difference images between any two images in different times by $R_{j}$ test statistic. (3) Segmenting the obtained difference images by the GSRM algorithm. (4) Modeling the segmented difference images by GGMM model, obtaining the statistical distribution of change and no-change classes. (5) Making decision analysis according to Equation (21) and calculating the change:

(21) $$CD(i,j)=\left\{ \begin{array}{l} 255,p(w_{u})p(x|w_{u})<p(w_{c})p(x|w_{c})\\ 0,\mathrm{otherwise} \end{array}\right.$$

where, ‘0’ represents no-change class and ‘255’ represents change classes.

## 3. Experiments and Results

### 3.1. Study Area and Background

**Figure 2:**
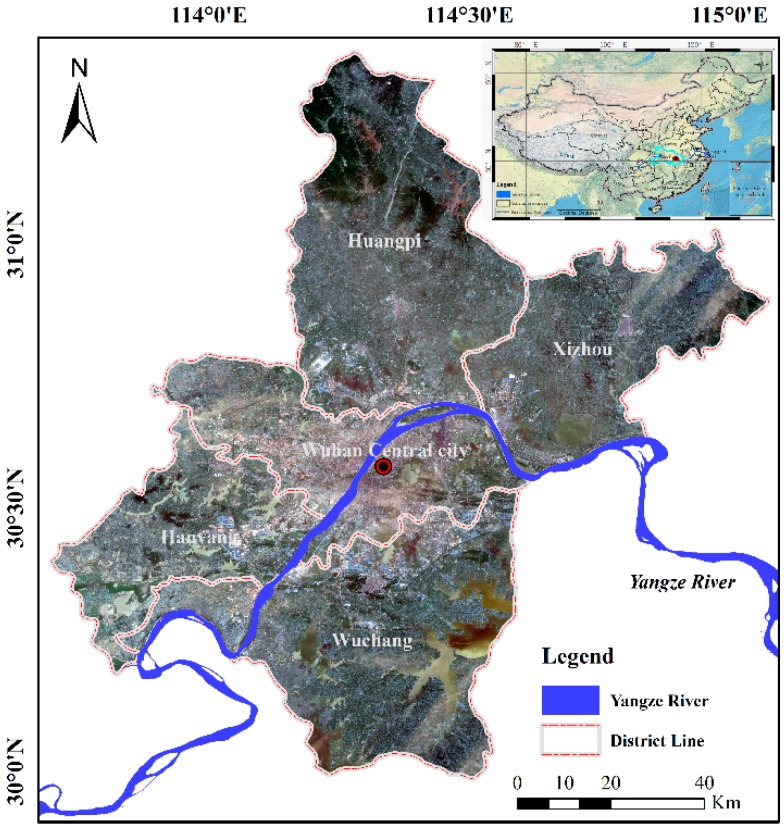
Location of the study areas.

The city of Wuhan (as shown in Figure 2) lies at East longitude 113°41′–115°05′, North latitude 29°58′–31°22′, and is the only megalopolis in the west of China. Wuhan is known as ‘River City’ as it is where the third largest river (Yangtze River) in the world and the largest tributary of the Yangtze River (Han River) converge. There are also many lakes in Wuhan, such as East Lake, LiangZi Lake and so on. Dramatic changes have taken place in the city of Wuhan during the ‘Twelfth Five-Year Plan’ period (from 2011 to 2015). Furthermore, the continuous heavy rain occurred in July 2016 and some areas showed dramatic change, especially LiangZi Lake. In order to detect the dramatic changes of city and flooded regions, time-series PolSAR images were acquired from the Radarsat-2 and GF-3 sensors. In this study, our aim was to detect the dramatic changes of city and flooded region (LiangZi Lake) and monitor the changes associated with the tunnel construction on East Lake. 

**Figure 3:**
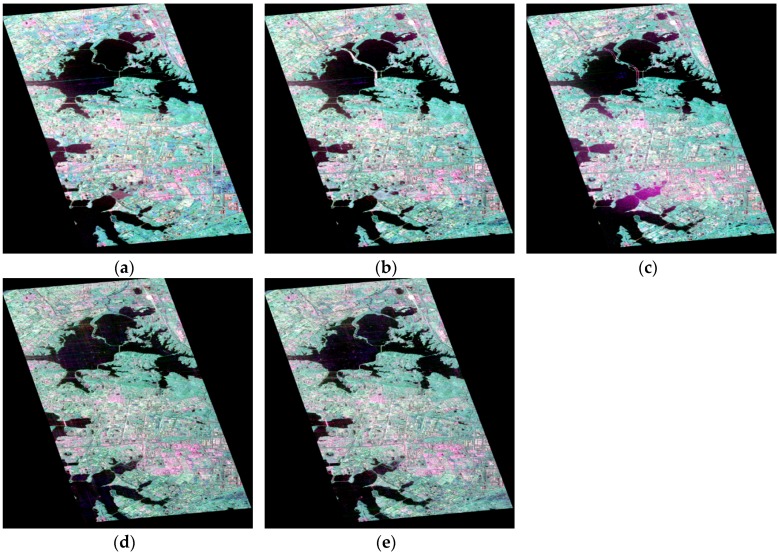
RADARSAT-2 PolSAR images acquired on (**a**) 7 December 2011; (**b**) 25 June 2015; and (**c**) 6 July 2016; and GF-3 PolSAR images acquired on (**d**) 30 April 2017; (**e**) 29 May 2017.

In order to detect the dramatic changes of the city, the C-band (single look complex) time-series full PolSAR images of Wuhan were acquired from Radarsat-2 and Gaofen-3 sensors and the parameters of the PolSAR images from the different sensors are shown in Table 2. The sizes of time-series PolSAR images are 2400 × 4200 pixels and the PauliRGB images are shown in Figure 3.

The preprocessing of PolSAR images is crucial to change detection when using time-series PolSAR images from different sensors. The pixel values are related to the backscatter from different PolSAR data, so the radiometric calibration of data is necessary. In particular, QualifyValue calibration of GF3 data is required using relevant parameters, which can be found in header file of GF3 data. Due to the different projection modes and spatial resolutions of different sensors, it is necessary to perform geometric correction and co-registration on the time-series PolSAR images using PolSARPro_v4.2.0 software and NEST software and the Root-Mean-Square Errors (RMSE) of co-registration were less than one pixel in this study. By comparing some filter methods of decreasing the influence of speckle noise, we found that the Lee Sigma filter has the better balance in the accuracy of results and less time-cost. Moreover, we also found that Lee Sigma filter could better retain details and preserve the shape of small land parcels when compare with other filters [33]. Therefore, a 7 × 7 Lee Sigma filter was chosen to decrease the influence of noise for the time-series PolSAR images.

### 3.2. Results

In order to assess the effectiveness of the proposed change detection algorithm, OT_GSRM_GGMM experiments were conducted with data from LiangZi Lake and East Lake. As mentioned above, omnibus test statistic algorithm is designed to generate the difference image over the entire time period, and $R_{j}$ test statistic is used to generate the difference images in any different time intervals.

#### 3.2.1. Omnibus Test Statistic of Time Series Change Detection over LiangZi Lake 

**Figure 4:**
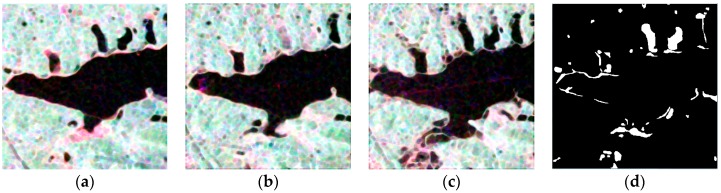

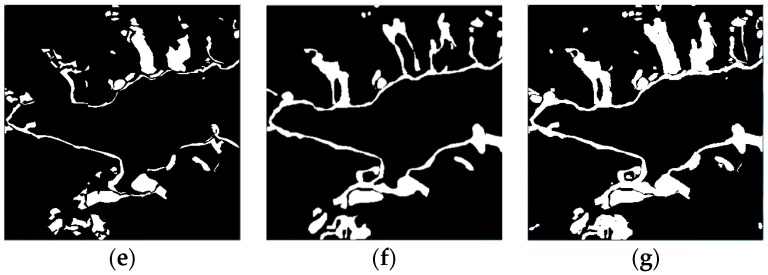
The Pauli-RGB images of LiangZi Lake after preprocessing on (**a**) 7 December 2011; (**b**) 25 June 2015; and (**c**) 6 July 2016; (**d**) Ground reference (2011–2015); (**e**) Ground reference (2015–2016); (**f**) Ground reference (2011–2016); (**g**) Ground reference (2011–2015–2016).

The datasets used in this research were time-series PolSAR images from Radarsat-2 acquired on 7 December 2011, 25 June 2015 and 6 July 2016, respectively. The Pauli-RGB images are shown in Figure 4a–c. Due to the restriction of natural conditions and observation data, we can only confirm the change caused by flooding on 6 July 2016, around LiangZi Lake. Meanwhile, other places and changes (such as the extension of the urban) in different time were obtained by visual interpretation of the optical imagery of GF-2 images corresponding to the time of PolSAR images. The ground references around LiangZi Lake are shown in Figure 4d–g. 

**Figure 5:**
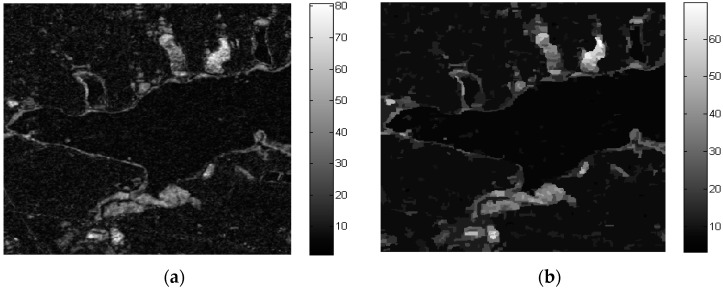
(**a**) The difference image based on omnibus test statistic; (**b**) The difference image after GSRM.

The overall difference image for LiangZi Lake was obtained by omnibus test statistic algorithm using time series (2011–2015–2016) PolSAR images and it is shown in Figure 5a. Affected by speckle noise, the difference image obtained by the traditional pixel-based change detection method still contains finely divided spots in Figure 5a. For this reason, the GSRM algorithm was used for segmentation, and the result after segmentation is shown in Figure 5b. The segmentation parameters are the scale parameter and gradient threshold, which were set to 32 and 0.5, respectively.

**Figure 6:**
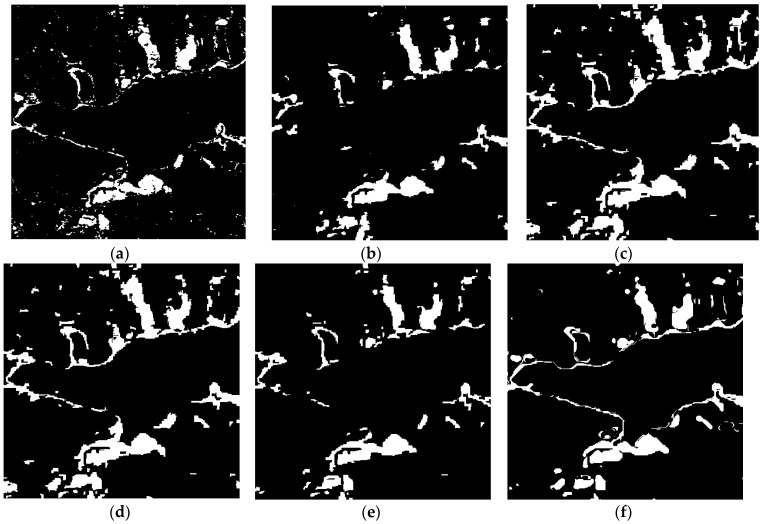
Change detection results of different algorithms over LiangZi Lake from 2011, 2015, and 2016. (**a**) Change detection result using OT_GGMM_pix (*k* = 22). (**b**) Change detection result using OT_GGMM_obj (*k* = 27). (**c**) Change detection results using OT_GSRM_TDES. (**d**) Change detection results using OT_GSRM_KI. (**e**) Change detection results using OT_GSRM_Kmeans. (**f**) Change detection results using the OT_GSRM_GGMM (*k* = 26). where, *k* is the optimal number of GMM.

From Figure 5b, it can be seen that the speckle noise can be suppressed using the GSRM algorithm. Different experiments ware undertake to verify the effectiveness of the proposed method: (1) comparison with the method of pixel-based using omnibus test statistic and GGMM algorithm (named OT_GGMM_pix) and it is shown in Figure 6a; (2) comparison with the method of object-based using omnibus test statistic and GGMM algorithm (named OT_GGMM_obj) and it is shown in Figure 6b; (3) comparison with the traditional method using TDES algorithm (named OT_GSRM_TDES) and it is shown in Figure 6c; (4) comparison with the traditional method using K&I algorithm (named OT_GSRM_KI) and it is shown in Figure 6d; (5) comparison with the traditional method using *k*-means algorithm (named OT_GSRM_Kmeans) and it is shown in Figure 6e. The white color represents change information and black color represents no-change information in change detection map. 

Comparing the results of different methods in Figure 6, due to the influence of speckle noise, divided spots can be found in the result of pixel-based change detection algorithm in the Figure 6a, which results in a higher *FA*. Object-oriented change detection algorithm can better suppress the speckle noise, but the method cannot maintain the details of the changes and a higher *OF* can be found in the Figure 6b. The proposed method not only can better suppress the speckle noise, maintain the details of the changes, but also improve the *OA* and *Kappa* when compare with traditional method (such as K&I, TDES, *k*-means algorithm) from the Table 3.

#### 3.2.2. *R_j_* Test Statistics of Time Series Change Detection over LiangZi Lake

**Figure 7:**
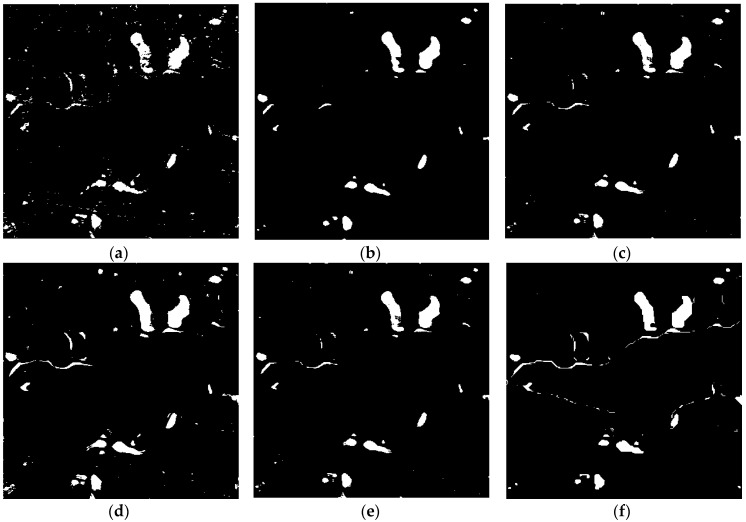
Change detection results of the different algorithms over LiangZi Lake between 2011 and 2015. (**a**) Change detection result using OT_GGMM_pix (*k* = 16). (**b**) Change detection result using OT_GGMM_obj (*k* = 14). (**c**) Change detection results using OT_GSRM_TDES. (**d**) Change detection results using OT_GSRM_KI. (**e**) Change detection results using OT_GSRM_Kmeans. (**f**) Change detection results using the OT_GSRM_GGMM (*k* = 21).

**Figure 8:**
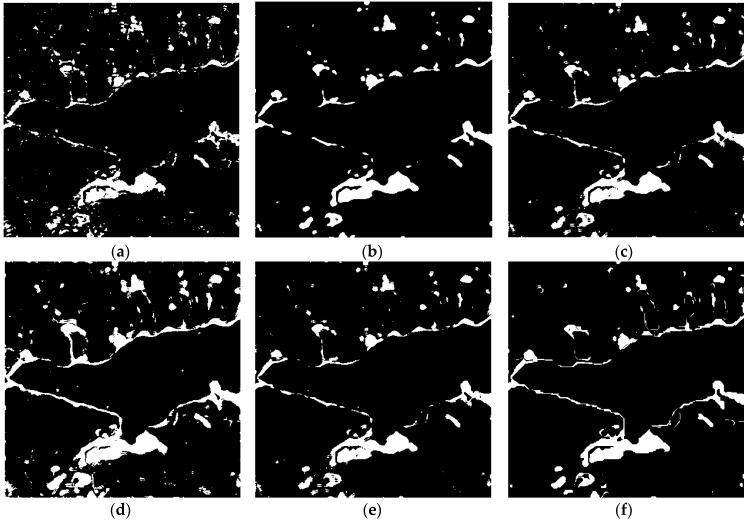
Change detection results of the different algorithms over LiangZi Lake between 2011 and 2016. (**a**) Change detection result using OT_GGMM_pix (*k* = 18). (**b**) Change detection result using OT_GGMM_obj (*k* = 14). (**c**) Change detection results using OT_GSRM_TDES. (**d**) Change detection results using OT_GSRM_KI. (**e**) Change detection results using OT_GSRM_Kmeans. (**f**) Change detection results using the OT_GSRM_GGMM (*k* = 22).

**Figure 9:**
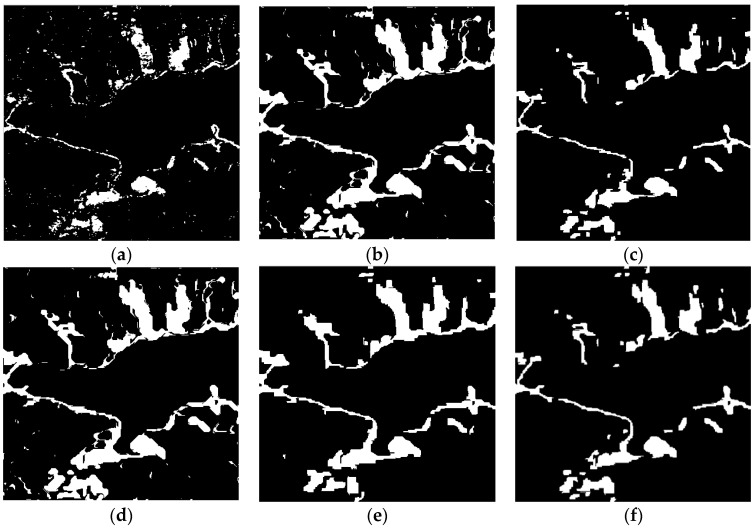
Change detection results of the different algorithms over LiangZi Lake between 2015 and 2016. (**a**) Change detection result using OT_GGMM_pix (*k* = 21). (**b**) Change detection result using OT_GGMM_obj (*k* = 33). (**c**) Change detection results using OT_GSRM_TDES. (**d**) Change detection results using OT_GSRM_KI. (**e**) Change detection results using OT_GSRM_Kmeans. (**f**) Change detection results using the OT_GSRM_GGMM (*k* = 25).

The above results obtained using the omnibus test statistic are binary and they can reflect the overall change from 2011 to 2016, however, they cannot explain when the change occurred. However, by combining *R_j_* test statistic processing, OT_GSRM_GGMM algorithm can detect the change between two different images in any time intervals. We therefore carried out three change detection experiments between two time intervals (2011 and 2015, 2011 and 2016, 2015 and 2016). The change detection results are shown in Figure 7, Figure 8 and Figure 9 respectively.

The results of change detection using different algorithms between 2011 and 2015 over LiangZi Lake are represented in Figure 7, where it can be seen that significant changes are detected. The *OA*, *FA*, *OF* and *Kappa* of different algorithms are listed in Table 4. The quantitative comparison of the six detection schemes indicates that the proposed approach shows a better performance than the other approaches as in the first experiment. 

We also compared and analyzed change detection results between 2011 and 2016, 2015 and 2016. Analogously, the results shown in Figure 8 and Figure 9 together with *OA*, *FA*, *OF* and *Kappa* (listed in Table 5 and Table 6). These results further confirm that the proposed method obtains better a change detection results.

**Figure 10:**
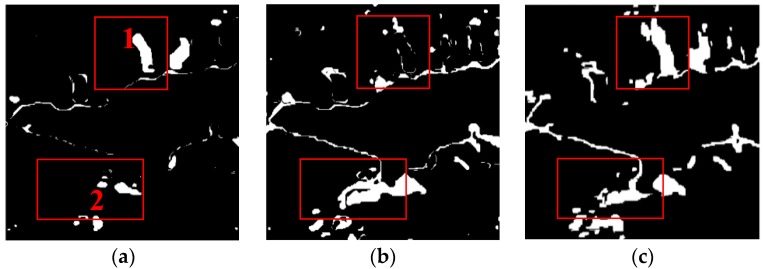
Change detection results (**a**) Between 2011 and 2015 using the OT_GSRM_GGMM. (**b**) Between 2011 and 2016 using the OT_GSRM_GGMM. (**c**) Between 2015 and 2016 using the OT_GSRM_GGMM.

Through a comprehensive comparison of omnibus test statistic and $R_{j}$ test statistic change detection results, it is clear that omnibus test statistic result (Figure 6f) reveals the overall change from 2011 to 2016, while $R_{j}$ test statistic results (Figure 10a–c) display the change for different locations and areas between two different times. Area 1 marked by a red rectangular box is a conspicuous change area between year 2011 and 2015. In the same location, the significant change also took place between 2015 and 2016 (Figure 10c), while there was little change between year 2011 and 2015. The reason for this is that the white part in area 1 was covered with water in year 2011 and 2016 and but was dry land in 2015. Moreover, from the white part of area 2 which identically marked by a red rectangular box, we can also see the different changes between different time intervals. Affected by the flooding in 2016, the change range between 2011 and 2016, 2015 and 2016 are larger than the change between 2011 and 2015. 

#### 3.2.3. Time-Series Change Detection over the East Lake Tunnel

**Figure 11:**
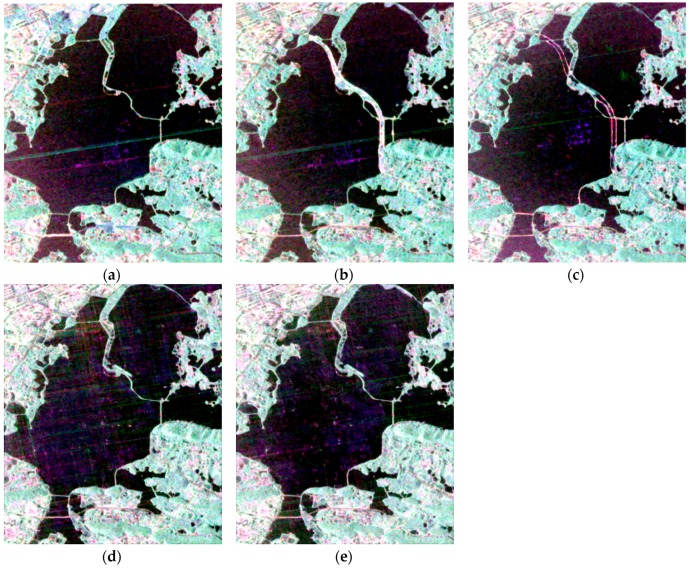
RADARSAT-2 PolSAR images acquired on (**a**) 7 December 2011. (**b**) 25 June 2015. (**c**) 6 July 2016; and GF-3 PolSAR images acquired on (**d**) 30 April 2017. (**e**) 29 May 2017.

The validity of the proposed algorithm applied to different sensors was verified. From 2011 to 2017, construction of a new tunnel and its ancillary buildings took place on East Lake in Wuhan, China. After the new tunnel was constructed, the ancillary buildings were removed in 2016. The land-cover types of this area are lake, bridge, city, and forest. PolSAR images acquired by Radarsat-2 and GF-3 sensors display the changes associated with the construction of the new tunnel on East Lake and the RGB images(1200 rows, 1000 columns) in Pauli basis are shown in Figure 11.

**Figure 12:**
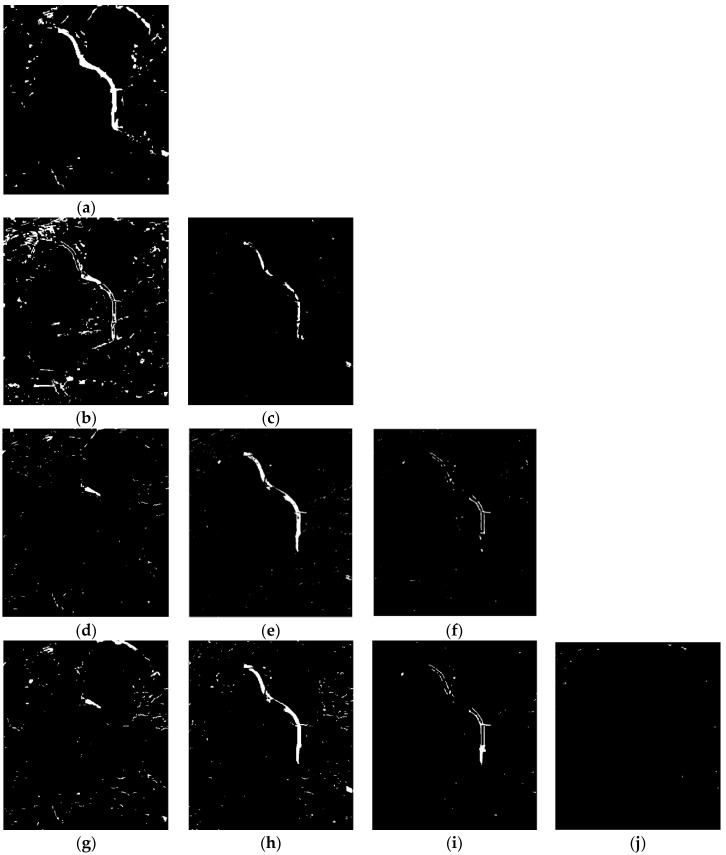
Time-series change detection over tunnel of East Lake from 2011 to 2017 (**a**) between December 2011 and June 2015. (**b**) Between December 2011 and July 2016. (**c**) Between June 2015 and July 2016. (**d**) Between December 2011 and April 2017. (**e**) Between June 2015 and April 2017. (**f**) Between July 2016 and April 2017. (**g**) Between December 2011 and May 2017. (**h**) Between June 2015 and May 2017. (**i**) Between July 2016 and May 2017. (**j**) Between April 2017and May 2017.

The proposed OT_GSRM_GGMM method was used to detect the changes in the construction process of East Lake tunnel. Dramatic changes took place around East Lake from 2011 to 2017 and the changes of different periods over East Lake are shown in Figure 12.

To give a visual impression of the results, Figure 12 shows the time-series change detection results over the East Lake tunnel from 2011 to 2017. Figure 12a shows the change detection result from December 2011 to June 2015, where the changes reflect the construction of the new tunnel and urbanization. Figure 12b shows the change detection result from 2011 to 2016, where the changes again reflect the construction of the new tunnel and urbanization. Figure 12c shows the change detection result from June 2015 to July 2016, where the changes reflect the removal of the ancillary buildings and urbanization. Figure 12d,g show the change detection results from December 2011 to April 2017 and from December 2011 to May 2017, where the changes reflect the urbanization. Figure 12e,h show the change detection results from December 2011 to April 2017 and from June 2015 to May 2017, respectively, where the changes reflect the removal of the ancillary buildings and urbanization. Figure 12f,i show the change detection results from July 2016 to April 2017 and from July 2016 to May 2017, where the changes reflect the removal of ancillary buildings. Figure 12j shows the change detection result from April 2017 to May 2017, where few changes can be observed.

## 4. Discussion 

Many existing unsupervised change detection methods using PolSAR images are limited by detecting change two images with different times from same sensor, and the results are subject to the influence of speckle noise. In this paper, focusing on the aforementioned problems of existing unsupervised change detection methods, we have proposed an automatic time-series unsupervised change detection approach (OT_GSRM_GGMM) using PolSAR images from different sensors. 

On the one hand, compared with the traditional methods of change detection, the proposed method has certain advantages:

(1) In the aspect of generation of the difference images, many different methods, such as the log-ratio operator, the hidden Markov chain model, and Kullback-Leibler divergence, were applied in multi-temporal single-channel SAR change detection and test statistics was applied in full PolSAR images. However, these traditional methods can only generate the difference image between two different times and they cannot generate the time-series difference images. Fortunately, omnibus test statistics can generate the difference image over the entire time series and *R_j_* test statistics can generate the difference images for the different time intervals in this paper.
(2) With regard to change detection analysis, some thresholding or clustering algorithms, such as k-means algorithm, fuzzy c-means algorithm, Otsu’s thresholding algorithms, Kapur’s entropy algorithms and K&I thresholding algorithm, can better analyze the difference image based on assumption that the PDF is Gaussian distributed for the changed and unchanged classes, but they are not suitable to analyze difference image of a non-Gaussian distribution. However, GGMM is capable of better fitting the arbitrarily conditional densities of the classes and it can also select the optimal number of components for the GMM in proposed method.
(3) In terms of image denoising, the object-oriented change detection algorithm can better suppress the influence of speckle noise, but some of the traditional segmentation algorithms cannot maintain the consistency between segmentation of time-series PolSAR images, the proposed method can avoid the inconsistency of segmentation by segmenting directly time-series difference images.

On the other hand, there still has some limitations in our proposed method:

(1) The design of our method is relatively complicated structure when compare with traditional methods of change detection.
(2) The experiment areas only chosen in urban and additional scenes, such as crop growing with different seasons, the change of suspended sediment concentration from different periods, were not considered yet.

## 5. Conclusions

To overcome the limitations of the existing unsupervised change detection methods, an unsupervised change detection method using time-series of PolSAR images was proposed in this paper, which integrates advantages of the omnibus test statistic, GSRM, and GGMM techniques in this paper. The omnibus test statistic algorithm was designed to detect the changes over the entire time period, and $R_{j}$ test statistics were used to detect changes in different time intervals. To suppress the influence of speckle noise, the GSRM algorithm was applied to segment difference images and GGMM was used to obtain time-series change detection maps. Using the proposed method, we were able to detect accurately the changes associated with the construction of a tunnel on East Lake from 2011 to 2017, dramatic changes in the city of Wuhan during ‘Twelfth Five-Year Plan‘ period, and also the changes of water bodies caused by the heavy rainfall in July 2016. The experimental results indicates that OT_GSRM_GGMM can not only detect small, gradual changes, but improve overall accuracy of the change detection result when compared with traditional unsupervised change detection methods. However, some future further improvements will still be necessary. For example, the computing efficiency can be improved by using the GPU technique and the time-series PolSAR images could be segmented using new segmentation techniques, such as watershed transform [34] and the level-set method [35]. Meanwhile, more experiments with additional scenes should be conducted and it also be of interest to try to detect seasonality in the changes. 

## Figures and Tables

**Table 1:** A summary of the traditional unsupervised change detection methods.

Methods	Relative Algorithms	Advantages	Disadvantages
Algebraic Operation	Image ratioing; Log-ratio operator; Regression analysis	Simple and easy to interpret change detection results.	Difficult to select the optimal threshold and easy to lose change information.
Transformation	Vegetation index differencing(VID); Change vector analysis (CVA); Principal component analysis (PCA); Tasselled cap transformation (KT)	Can reduce the data redundancy and also emphasize the different information of the derived components.	Strictly require the remotely sensed data acquired from the same phenological period and difficult to select the optimal threshold.
Object-based change detection	Direct object-basedcomparison	Allow straight forward comparison of objects and reduce the influence of speckle noise.	Some of the traditional segmentation algorithms cannot maintain the consistency of PolSAR images and might cause higher false alarm rates.
Other methods	Hidden Markov chain model (HMM); Kullback-Leibler divergence and so on.	Simple and can be applied in multi-temporal single-channel SAR change detection.	Cannot be applied in multi-temporal PolSAR change detection and the selection of thresholding based on a Gaussian distribution.

**Table 2:** The parameters of the Radarsat-2 and Gaofen-3 images.

Acquisition Date	Sensors	Mode	Processing Level	Polarization	ProductId
7 December 2011	Radarsat-2	FQ21	Single Look Complex	HH + HV + VH + VV	RD2011000503-0
25 June 2015	Radarsat-2	FQ21	Single Look Complex	HH + HV + VH + VV	PDS_04516040
6 July 2016	Radarsat-2	FQ21	Single Look Complex	HH + HV + VH + VV	PDS_05215280
30 April 2017	Gaofen-3	QPSI	Single Look Complex	HH + HV + VH + VV	2335427
29 May 2017	Gaofen-3	QPSI	Single Look Complex	HH + HV + VH + VV	2390686

**Table 3:** Performance evaluation over LiangZi Lake from 2011, 2015, and 2016.

Method	*OA*	*FA*	*OF*	*Kappa*
OT_GGMM_pix	89.91%	1.43%	8.65%	0.54
OT_GGMM_obj	91.03%	1.13%	7.83%	0.59
OT_GSRM_KI	92.35%	1.73%	5.91%	0.70
OT_GSRM_TDES	92.29%	2.22%	5.70%	0.69
OT_GSRM_Kmeans	90.87%	0.32%	8.80%	0.59
OT_GSRM_GGMM	93.85%	0.27%	6.87%	0.71

**Table 4:** Performance evaluation over LiangZi Lake between 2011 and 2015.

Method	*OA*	*FA*	*OF*	*Kappa*
OT_GGMM_pix	94.64%	2.43%	2.93%	0.66
OT_GGMM_obj	95.55%	2.04%	2.41%	0.69
OT_GSRM_KI	95.14%	2.23%	2.63%	0.68
OT_GSRM_TDES	95.16%	2.13%	2.71%	0.68
OT_GSRM_Kmeans	95.20%	2.31%	2.49%	0.68
OT_GSRM_GGMM	96.98%	1.29%	1.73%	0.78

**Table 5:** Performance evaluation over LiangZi Lake between 2011 and 2016.

Method	*OA*	*FA*	*OF*	*Kappa*
OT_GGMM_pix	92.46%	1.23%	6.29%	0.58
OT_GGMM_obj	94.03%	0.57%	5.38%	0.65
OT_GSRM_KI	93.16%	0.82%	6.01%	0.61
OT_GSRM_TDES	93.29%	0.75%	5.95%	0.62
OT_GSRM_Kmeans	93.28%	0.75%	5.96%	0.62
OT_GSRM_GGMM	95.95%	0.60%	3.44%	0.71

**Table 6:** Performance evaluation over LiangZi Lake between 2015 and 2016.

Method	*OA*	*FA*	*OF*	*Kappa*
OT_GGMM_pix	95.12%	2.07%	2.97%	0.70
OT_GGMM_obj	95.41%	1.90%	2.74%	0.73
OT_GSRM_KI	95.30%	3.51%	1.17%	0.74
OT_GSRM_TDES	95.49%	2.12%	2.38%	0.74
OT_GSRM_Kmeans	94.29%	4.58%	1.12%	0.71
OT_GSRM_GGMM	96.22%	1.56%	2.20%	0.76
